# Pilot study about the incidence of missed injuries after implantation of protocol of tertiary survey in patients with severe trauma

**DOI:** 10.1186/2197-425X-3-S1-A371

**Published:** 2015-10-01

**Authors:** S Chacón Alves, H Marín Mateos, G Morales Varas, M Chico Fernández, C García Fuentes, D Toral Vázquez, S Bermejo Aznárez, L Terceros Almanza, E Alted López, JC Montejo González

**Affiliations:** Medicina Intensiva, UCI de Trauma y Emergencias, Hospital Universitario Doce de Octubre, Madrid, Spain

## Introduction

Despite of primary and secondary survey in patients with severe trauma, missed injuries can occur, so some authors consider that the tertiary survey could reduce its incidence.

## Objectives

Protocol of tertiary trauma survey was established after literature review, to detect missed injuries in the initial care and as part of the Quality program Unit.

## Methods

Prospective, descriptive, observational study, in patients with severe trauma (Injury Severity Score (ISS) > 15), who were admitted to the Trauma and Emergency ICU of a high complexity hospital from May 2013 to January 2014. The tertiary survey was performed at 24-48 hours of admission patient and it was conducted by an experienced staff and a resident not involved in the initial patient care. We collected demographic variables, initial Glasgow Coma Score (GCS), Injury Severity Score, hemodynamic status on admission, length of stay in ICU, days of Mechanical Ventilation (MV), complications and mortality. Furthermore, we collected missed injuries and their impact on patients outcomes. Quantitative variables were expressed as mean ± standard deviation. For qualitative variables we used percentages. Statistical data were analyzed by SPSS 16.0 considering statistically significant P < 0.05.

## Results

88 patients were studied, 72.7% male, mean age of 40.63 ± 17.71, average ISS was 22.02 ± 11.74, and GCS ≤ 8 21.8%. The median ICU stay was 2.88 days (IQR, 1.48-8.12), with 3.42 ± 3.39 days connected to MV. Overall mortality of 4%. 45 missed injuries were found in 34 patients (38.6% of all patients). There were: extremities and soft tissue injuries (44.4%), head and neck injuries (24.4%) and back injuries (17.8%). 55.6% of them had no impact on patients outcomes, 22.2% required a new complementary test and none of them caused the patient´s death. The presence of a higher ISS, was associated with significantly greater number of missed injuries (p < 0.05).

22 new radiological findings in 20 patients were diagnosed: 56.5% were fractures, the majority in costal arches. 72.7% of missed diagnosis had no impact on the patients evolution, 13.6% required a new specialist evaluation and none of them caused the death.

## Conclusions

These findings match with the published literature about incidence of missed injuries and demonstrate the importance of establishing a tertiary trauma survey for early identification and as a quality indicator. The presence of a higher ISS could be considered a risk factor for these lesions.
